# Factors Affecting Mortality and Clinical Outcomes in Intensive Care Unit Patients with Thoracic Trauma: A Retrospective, Single-Center Study

**DOI:** 10.3390/medicina62020294

**Published:** 2026-02-02

**Authors:** Yeşim Şerife Bayraktar, Tuba Şahinoğlu, Yasemin Cebeci, Dilara Cari Güngör, Büşra Pekince, Muslu Kazım Körez, Atilla Can, Jale Bengi Çelik

**Affiliations:** 1 Division of Intensive Care, Department of Anesthesiology and Reanimation, Selçuk University Faculty of Medicine, Konya 42130, Turkey; jalecelik@hotmail.com; 2 Department of Thoracic Surgery, Selçuk University Faculty of Medicine, Konya 42130, Turkey; tkilicer@yahoo.com (T.Ş.); atillacan_ac@yahoo.com (A.C.); 3 Division of Intensive Care, Department of Anesthesiology and Reanimation, Beyhekim Training and Research Hospital, Konya 42060, Turkey; dryasemin.cebeci@gmail.com; 4 Division of Intensive Care, Department of Neurology, Konya City Hospital, University of Health Science, Konya 42020, Turkey; dcarigungor@hotmail.com; 5 Division of Intensive Care, Department of Pulmonology, Konya Numune Hospital, Konya 42060, Turkey; dr.bustek@gmail.com; 6 Department of Biostatistics, Selçuk University Faculty of Medicine, Konya 42130, Turkey; mkkorez@gmail.com

**Keywords:** mortality, thoracic injuries, trauma, intensive care units, APACHE II, lactate, acidosis

## Abstract

*Background and Objectives*: Thoracic trauma usually results in high morbidity and mortality. It is the leading cause of death in patients within the first four decades of life. In this study, we aimed to identify risk factors for intensive care mortality and to evaluate factors affecting clinical outcomes and complications in patients with thoracic trauma who were treated in the intensive care unit (ICU). *Materials and Methods*: This was a retrospective, single-center study. Patients diagnosed with thoracic trauma and followed up in the ICU between 1 May 2023 and 1 January 2025 were included. Critically ill patients aged 18 years and older whose admission blood values were available and who had undergone radiological imaging were included in the study. Patients were grouped as Survivors or Non-survivors. The primary outcome was to determine risk factors for mortality. The secondary outcome was to evaluate factors affecting clinical outcomes and complications. The tertiary outcome was to determine the predictive value of the Injury Severity Score (ISS), Acute Physiology and Chronic Health Evaluation II (APACHE II), and Glasgow Coma Scale (GCS) for mortality. *Results*: A total of 104 patients (male/female ratio: 76/28) were included in the study. Twenty-four patients (23.1%) died, and eighty (76.9%) were discharged. Age in the Non-survivor group was found to be significantly higher (59.33 ± 22.21 vs. 40.50 ± 17.71; *p* < 0.001), and the proportion of women was also significantly higher in the Non-survivor group (*p* = 0.0082). Mortality was associated with advanced age, female sex, lower GCS score (*p* < 0.001), higher APACHE II scores (*p* < 0.001), and the presence of comorbid conditions (*p* = 0.003), including head trauma (*p* = 0.024) and cardiac arrest before ICU admission (*p* = 0.011). The Non-survivor group more frequently required mechanical ventilation (*p* < 0.001), vasopressor support (*p* < 0.001), and continuous renal replacement therapy (*p* < 0.001), and they developed ventilator-associated pneumonia (*p* < 0.001) and acute respiratory distress syndrome (*p* < 0.001) at higher rates. ICU length of stay was also significantly longer in the Non-survivor group (*p* = 0.045). The APACHE II score demonstrated the highest discriminatory performance, emerging as the strongest clinical predictor of mortality (AUC = 0.751, 95% CI: 0.630–0.872; *p* < 0.001). Age (OR: 1.06) and serum lactate levels (OR: 1.57) consistently emerged as strong independent predictors of mortality. The presence of head trauma significantly increased the risk of mortality, particularly in the APACHE II-adjusted model (OR: 9.08). The APACHE II–based model yielded high specificity (96.3%) and accuracy (88.5%), with good discrimination (AUC = 0.894) and the highest Nagelkerke R^2^ (0.548). *Conclusions*: Factors that may shorten the length of ICU stay include infection control, early correction of acidosis, and maintenance of hemodynamic stability, which may reduce mortality. APACHE II was more closely related to overall clinical severity than the other scoring systems. Our data indicate that age-related frailty and acute physiological derangement, as best represented by the APACHE II score, are more significant determinants of survival than anatomic injury severity alone.

## 1. Introduction

Thoracic trauma usually results in high morbidity and mortality. It is the leading cause of death in patients within the first four decades of life. Thoracic trauma is the second most common traumatic injury resulting from accidents, contributing most to mortality on a global scale. It accounts for approximately 10–15 per cent of all trauma cases [1]. It accounts for approximately 25 to 35 per cent of trauma-related deaths [1,2].

Thoracic injuries occur after penetrating and blunt injuries. Blunt chest trauma is more common than penetrating trauma (70–80%) and accounts for 20 to 25% of trauma deaths [3].

Within the first hour after hospital admission, thoracic vascular and neurological trauma are the most common causes of death [4]. In critically ill patients, rapid diagnosis and prompt implementation of effective and aggressive treatment are essential. Intensive care units (ICUs) are facilities where appropriate treatment is administered and vital signs are closely monitored. Identifying risk factors in patients admitted to intensive care with thoracic trauma is of vital importance for their clinical course, outcomes, and complications. It is crucial for determining appropriate treatment protocols. However, patients requiring intensive care represent a distinct subgroup with greater physiological instability and a higher burden of complications, in whom outcome determinants may differ from those of the general thoracic trauma population. Unlike the general trauma population, ICU-admitted thoracic trauma patients exhibit greater physiological instability, a higher burden of multi-organ involvement, and an increased susceptibility to secondary complications such as acute respiratory distress syndrome (ARDS) and ventilator-associated pneumonia (VAP) [5]. There is a great deal of research on general trauma patients, but the mortality predictors for thoracic trauma patients monitored in ICUs remain unclear at the local level. Identifying these local risk factors is vital for improving triage protocols and optimizing resource allocation.

In this study, we primarily aimed to identify risk factors for intensive care mortality and to evaluate factors affecting clinical outcomes and complications, including intensive care unit length of stay, pneumonia, vasopressor requirement, and continuous renal replacement therapy (CRRT) requirement, in patients with thoracic trauma who were followed up and treated in our intensive care unit. Our secondary aim was to determine the predictive value of established scoring systems, such as the Injury Severity Score (ISS), the Acute Physiology and Chronic Health Evaluation II (APACHE II), and the Glasgow Coma Scale (GCS), within this specific population.

## 2. Materials and Methods

### 2.1. Study Design and Setting

Given the importance of identifying local mortality predictors, we designed a cohort study in which patients with thoracic trauma who were treated at the intensive care unit (ICU) of Selçuk University Faculty of Medicine between 1 May 2023 and 1 January 2025 were retrospectively evaluated. The research protocol complied with the Declaration of Helsinki. Approval was obtained from the local Ethics Committee (Selçuk University Faculty of Medicine Ethics Committee, Konya, Turkey, approval number: 2025/146; date: 13 March 2025). Data was collected from the hospital’s validated electronic health record system via a standardized case report form to reduce data entry anomalies. To verify the reliability and integrity of the dataset, a dual-verification method was implemented: two independent researchers checked the digital records, and any differences were handled by a third senior doctor. Furthermore, all laboratory results and physiological parameters were received immediately from the automated laboratory information system, which avoids the subjectivity sometimes associated with manual chart reviews.

### 2.2. Participants

#### 2.2.1. Inclusion Criteria

Patients diagnosed with thoracic trauma and followed up and treated in the ICU were included. Critically ill patients aged 18 years and older whose admission blood values from routine examinations were available in full and who had undergone radiological imaging were included in the study. The diagnosis of thoracic trauma was made based on chest CT and/or chest X-ray images [6].

#### 2.2.2. Exclusion Criteria

Individuals under the age of 18, pregnant patients, and those who died within the first 24 h were excluded from the study. Those with incomplete routine examination results taken during hospitalization were excluded from the study.

### 2.3. Study Design

The demographic characteristics of the patients and their comorbidities (Diabetes Mellitus, cerebrovascular disease, cardiac disease, chronic kidney disease, hypertension, malignancy) were recorded.

The presence of head trauma was recorded. It was recorded whether the injury was blunt or penetrating. Pneumothorax, hemothorax, pneumomediastinum, pulmonary contusion, scapular fracture, clavicle fracture, sternal fracture, rib fracture, and skeletal system pathologies were recorded from the patient’s radiological imaging and records. It was noted whether a thoracic surgery consultation was performed and the content of the consultation notes. Those who underwent surgical operations for the thorax and those who underwent surgery for other injuries were recorded. Those who underwent tube thoracostomy were recorded.

The Injury Severity Score (ISS), the Acute Physiology and Chronic Health Evaluation II (APACHE II), and the Glasgow Coma Scale (GCS) were administered upon admission to intensive care. The severity of the injury was graded using the ISS [7], and the severity of head trauma was graded using the GCS [8]. The APACHE II score, routinely calculated upon admission to the ICU and serving as a mortality indicator, was recorded [9].

Whether cardiac arrest had occurred prior to intensive care admission and whether the patient was intubated or breathing spontaneously were recorded. Traumatic cardiac arrest (TCA) is a common term used to describe circulatory arrest following major trauma [10]. Whether patients had TCA prior to intensive care admission was recorded.

Laboratory tests (hemogram, platelet count, urea, creatine, calcium, prothrombin time (PT), partial thromboplastin time (PTT), INR, and arterial blood gas values) taken during routine examinations on the day of admission to intensive care were recorded. Patients were grouped as Survivors or Non-survivors. Factors affecting clinical outcomes, including length of stay in intensive care, pneumonia, CRRT requirement, vasopressors requirement, mechanical ventilation (MV) requirement, MV time, and duration of ICU stay, were recorded for both groups.

Aspiration pneumonia (ASP) was diagnosed following confirmation of inflammatory findings in the lungs and the presence of overt aspiration or dysphagia [11]. Ventilator-associated pneumonia (VAP) was diagnosed as a nosocomial infection of the lung parenchyma occurring after 48 h of tracheal intubation and mechanical ventilation [12]. The development of ASP and VAP was recorded in patients.

The primary outcome was to determine the risk factors for intensive care mortality. The secondary outcome was to evaluate factors affecting clinical outcomes and complications, including length of stay in intensive care, pneumonia, development of ARDS, vasopressor requirement, and CRRT requirement.

The tertiary outcome was to determine the predictive value of ISI, APACHE II, and GCS, commonly used in trauma patients to assess prognosis and clinical course, for mortality.

### 2.4. Statistical Analysis

All statistical analyses were performed using R version 4.5.2 (www.r-project.org). Prior to analysis, data normality was assessed using the Shapiro–Wilk normality test, and group variance homogeneity was assessed using the Levene test. Numerical variables were summarized as mean ± standard deviation, median (minimum–maximum) or median with quartiles [first quartile–third quartile], depending on their suitability, while categorical variables were described as frequency (n) and percentage (%). The demographic data of surviving and deceased patients, their clinical characteristics at admission, trauma and thoracic injury profiles, laboratory parameters, and complications related to the intensive care process were compared using Student’s *t*-test, Welch’s *t*-test, Mann–Whitney U test, or chi-square tests. The discriminatory power of three clinical scores (Glasgow Coma Scale, Injury Severity Score, and APACHE II) used to predict mortality in patients with thoracic trauma monitored in intensive care was evaluated using ROC curve analysis. The area under the curve (AUC), sensitivity, specificity, ppv, and npv values were calculated with 95% confidence intervals. The significance level was set at 5%. A multivariate logistic regression analysis was performed to determine the relationship between clinical scores and mortality in patients with thoracic trauma admitted to the intensive care unit. The analysis results were reported as adjusted odds ratios (ORs) and 95% confidence intervals (CIs). The model was adjusted for age, gender, serum lactate levels, and the presence of head trauma.

## 3. Results

During the study period (1 May 2023 and 1 January 2025), 1642 patients were admitted to our ICU. Of these, 111 had thoracic trauma. Seven pediatric patients were excluded. Thus, 104 patients were included in the study. Twenty-four patients (23.1%) died, and eighty (76.9%) were discharged.

Table 1 presents a comparative analysis of the demographic data, clinical characteristics at presentation, trauma and thoracic injury profiles, and laboratory parameters of patients who did not survive (Non-survivors) and those who were discharged from the ICU (Survivors). The age of the Non-survivor group was found to be significantly higher (59.33 ± 22.21 vs. 40.50 ± 17.71; *p* < 0.001), and the proportion of women was also significantly higher in the Non-survivor group (*p* = 0.0082). When clinical scores at admission were examined, the GCS score was significantly lower in the Non-survivor group (6 vs. 15; *p* < 0.001), while the APACHE II score was significantly higher (23 vs. 12; *p* < 0.001). The ISS was similar in both groups but was not statistically significant (*p* = 0.075).

Injuries accompanying trauma, including head trauma, were significantly more common in the Non-survivor group (83.3% vs. 55%; *p* = 0.024). The presence of additional diseases was also strongly associated with mortality (37.5% vs. 10%; *p* = 0.003). Cardiac disease (*p* = 0.038) and hypertension (*p* = 0.015) were particularly higher in the Non-survivor group. Pre-admission cardiac arrest was only observed in the Non-survivor group and was found to be significant (*p* = 0.011) (Table 1).

Although the two groups were generally similar in terms of thoracic injuries, sternal fractures were more common in the Non-survivor group and showed statistical significance at the threshold (*p* = 0.065). Apart from this, injuries such as pneumothorax, hemothorax, and pulmonary contusion were not found to be associated with mortality (Table 1).

Upon examination of laboratory findings, it was observed that, in the Non-survivors group, hemoglobin levels were lower (*p* < 0.001) and urea (*p* < 0.001), creatine (*p* = 0.002), INR (*p* < 0.001), and lactate levels were higher, while pH was lower (*p* < 0.001). These findings support more severe metabolic and organ dysfunction in the Non-survivor group. The more negative base deficit (BE) value (*p* = 0.003) also reflects marked acidosis in the Non-survivors group (Table 1).

Table 2 shows a comparative overview of the course, outcomes, and complications of the intensive care process for patients who did not survive (Non-survivors) and those who discharged from the ICU (Survivors). Complications and the clinical course during intensive care were strongly associated with mortality. The need for mechanical ventilation (95.8% vs. 30%; *p* < 0.001), development of VAP (58.3% vs. 16.3%; *p* < 0.001), need for CRRT (29.2% vs. 2.5%; *p* < 0.001), vasopressor requirement (75% vs. 28.8%; *p* < 0.001), and development of ARDS (29.2% vs. 2.5%; *p* < 0.001) were significantly more frequent in the Non-survivor group. The ICU length of stay was also significantly longer in the mortality group (*p* = 0.045).

**Figure 1:**
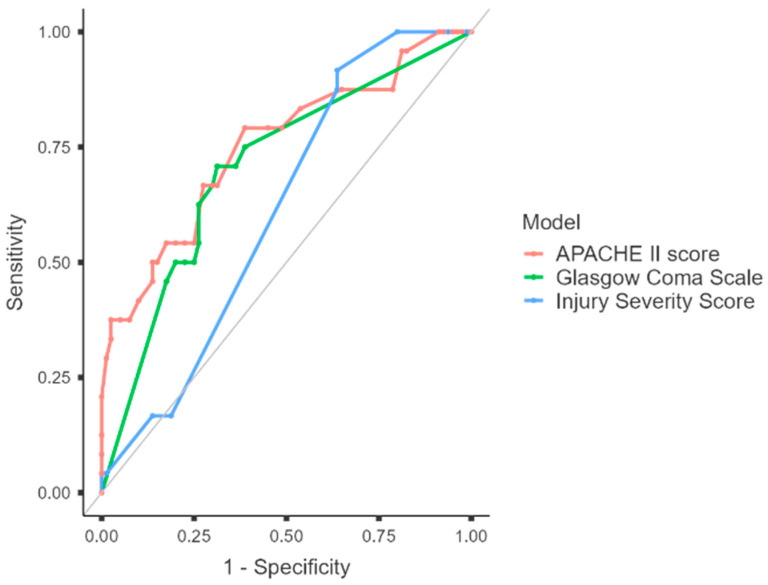
ROC curves for Acute Physiology and Chronic Health Evaluation II (APACHE II), Glasgow Coma Scale, and Injury Severity Score for mortality following thoracic trauma.

The discriminatory power of three clinical scores (GCS, ISI, and APACHE II) used to predict clinical course and mortality in patients with thoracic trauma monitored in the ICU was evaluated using ROC curve analysis (Figure 1). The APACHE II score demonstrated the highest discriminatory performance among all indicators, emerging as the strongest clinical predictor of mortality (AUC = 0.751, 95% CI: 0.630–0.872; *p* < 0.001). The optimal cut-off point according to the Youden index was determined to be ≥15. At this threshold, 79.17% sensitivity and 61.25% specificity were achieved. Although the positive predictive value was limited (38.0%), the high negative predictive value (90.74%) indicates that low APACHE II scores are a reassuring indicator in terms of mortality (Table 3).

The GCS has been found to have moderate discriminatory power as a parameter reflecting the severity of neurological status (AUC = 0.706, 95% CI: 0.592–0.820; *p* < 0.001). The optimal threshold value was determined to be ≤12; at this point, sensitivity was calculated as 70.83% and specificity as 68.75%. Despite the low PPV (40.48%), the high NPV (88.71%) indicates that the probability of survival is significantly higher in patients with good neurological reserve (Table 3).

The ISS demonstrated the lowest performance in predicting mortality (AUC = 0.612, 95% CI: 0.512–0.712; *p* = 0.028). With a threshold value of ≥25, sensitivity was found to be quite high (91.67%); however, specificity was markedly low (36.25%). Therefore, although ISS is valuable for not missing high-risk patients in the early stages due to its high sensitivity, it is insufficient for confirming high-risk patients on its own (Table 3).

Table 4 presents the results of multivariable logistic regression analyses evaluating the association between clinical scores and mortality among thoracic trauma patients admitted to the ICU, after adjustment for major clinical confounders including age, sex, serum lactate levels, and the presence of head trauma.

In **Model 1**, which included age, sex, lactate, head trauma, and the Glasgow Coma Score (GCS), increasing age was independently associated with higher mortality risk (OR = 1.07, 95% CI: 1.03–1.11; *p* < 0.001). Elevated serum lactate levels also remained a significant predictor of mortality (OR = 1.64, 95% CI: 1.12–2.39; *p* = 0.011). The presence of head trauma was associated with a markedly increased risk of death (OR = 5.45, 95% CI: 1.14–26.06; *p* = 0.034). GCS demonstrated a borderline protective effect (OR = 0.89, 95% CI: 0.79–1.00; *p* = 0.056), whereas sex was not independently associated with mortality. This model showed strong overall performance, with high accuracy (90.4%), excellent specificity (96.3%), acceptable sensitivity (70.8%), and good discrimination (AUC = 0.887). The Nagelkerke R^2^ was 0.544, indicating substantial explanatory power.

In **Model 2**, where the GCS was replaced by the Injury Severity Score (ISS), age (OR = 1.07, 95% CI: 1.03–1.12; *p* < 0.001) and lactate (OR = 1.89, 95% CI: 1.26–2.83; *p* = 0.002) remained independent predictors of mortality. Although head trauma and ISS showed positive associations with mortality risk, these did not reach statistical significance. This model achieved the highest discriminatory performance among all evaluated models (AUC = 0.898), with an accuracy of 85.6% and a Nagelkerke R^2^ of 0.538. The AIC value (78.73) indicated a competitive model fit.

The final model (**Model 3**), incorporating the APACHE II score instead of the ISS, demonstrated that age (OR = 1.06, 95% CI: 1.02–1.10; *p* = 0.002), lactate (OR = 1.57, 95% CI: 1.05–2.34; *p* = 0.027), and head trauma (OR = 9.08, 95% CI: 1.75–47.16; *p* = 0.009) were independently associated with increased mortality risk. Notably, the APACHE II score was also an independent predictor of mortality (OR = 1.07, 95% CI: 1.00–1.15; *p* = 0.045). This model yielded high specificity (96.3%) and accuracy (88.5%), with good discrimination (AUC = 0.894) and the highest Nagelkerke R^2^ (0.548). Moreover, it demonstrated the lowest AIC value (77.61), suggesting the best overall balance between model fit and complexity.

Overall, across all models, age and serum lactate levels consistently emerged as robust independent predictors of mortality in thoracic trauma patients. The presence of head trauma substantially amplified mortality risk, particularly in the APACHE II–adjusted model. Among the evaluated severity scoring approaches, the APACHE II–based model provided the most favorable balance between explanatory power and model fit, while ISS- and GCS-based models offered comparable discriminatory performance with differing sensitivity–specificity trade-offs.

## 4. Discussion

In this study, the all-cause mortality rate among patients with thoracic trauma followed in the ICU was 23.1%, which is in line with rates reported in comparable ICU-based trauma cohorts [2,6]. Mortality was associated with advanced age, female sex, lower GCS score at admission, higher APACHE II scores, the presence of comorbid conditions, accompanying head trauma and traumatic cardiac arrest before ICU admission. Patients who did not survive also had a more challenging intensive care course. They more frequently required mechanical ventilation, vasopressor support, and continuous renal replacement therapy, and they developed ventilator-associated pneumonia and acute respiratory distress syndrome at higher rates. Taken together, these observations indicate that outcomes in ICU-managed thoracic trauma patients are influenced not only by the severity of thoracic injury, but also by physiological reserve, accompanying systemic insults, and complications arising during intensive care follow-up.

Elderly patients who have suffered chest trauma may experience worse outcomes due to frailty and other comorbidities [3]. In our study, advanced age and comorbid conditions, particularly hypertension and cardiac disease, were more common in patients who died. These conditions may reduce the ability to tolerate both the initial trauma and the subsequent intensive care process. Our findings suggest that mortality is not determined by age alone but rather by the combined effect of age and underlying systemic disease. Age and lactate levels are consistently identified as independent risk factors in all of our models, indicating their strong predictive value. These results show that, regardless of damage pattern, biochemical perfusion measures and increased age-related frailty in patients receiving thoracic trauma follow-up are independent predictors of mortality.

Traumatic cardiac arrest (TCA) is associated with a high mortality rate ranging from 92.3% to 100% [10]. However, an epidemiological study conducted in Sweden reported a 30-day survival rate of 10.6 per cent [10,13]. In our study, traumatic cardiac arrest was observed only in patients who did not survive and was associated with mortality. The number of patients was small; however, this finding is consistent with the clinical experience, suggesting that cardiac arrest following major trauma usually indicates severe physiological deterioration. Even with intensive care support, outcomes in these patients remain poor.

Previous studies have reported that the most frequently reported associated injury in patients with thoracic trauma is head trauma [14]. In a study by Küçük et al. involving 564 patients, mortality was found to be significantly higher only in those with accompanying head trauma [6]. Lin et al. reported that cases of thoracic trauma accompanied by head injury required intensive care and remained in intensive care for longer than most other cases of thoracic trauma [14]. In our study, head trauma was also more frequently associated with the Non-survivor group. The intensive care admission period was also found to be significantly longer. However, ARDS, VAP, the need for mechanical ventilation, and the presence of additional diseases were also found to be significantly associated with mortality in the Non-survivor group. For this reason, head trauma alone cannot explain mortality, but it likely adds to the overall severity of the clinical course. Our multivariate study revealed that head trauma raises the risk of death by 9.08 times (OR) when evaluated in conjunction with APACHE II, despite the fact that head trauma alone is not a predictor. This evidence demonstrates that when head trauma is coupled with other systemic disorders, the clinical course is significantly worsened.

In a retrospective study of thoracic trauma patients, Singh et al. found that age, number of rib fractures, admission lasting longer than 24 h, and the need for mechanical ventilation were strongly associated with mortality [1]. Age and the need for mechanical ventilation were similarly high in the Non-survivor group in our study.

Mechanical ventilation is a frequently used and vitally important treatment method in intensive care. However, prolonged mechanical ventilation can lead to an increased risk of infection and complications. A common complication of mechanical ventilation is VAP [15]. A retrospective study conducted in 2017 reported that ventilation duration and ICU stay duration in patients with VAP were significantly longer than in patients without VAP. VAP significantly increases hospital stay duration and healthcare costs, and it is also associated with long-term morbidity and mortality [12]. An increased need for mechanical ventilation [15], as is the case with VAP, is also a risk factor for ARDS. The global definition of ARDS has recently been published [16]. It has been noted that trauma itself is a risk factor for pulmonary edema [16].

In patients with thoracic trauma treated in the ICU, the main determining factors for prolonged mechanical ventilation include the presence of bilateral chest injuries, age, and the degree of neurotrauma [14].

Most chest injuries can be treated with simple interventions such as tube thoracostomy; however, 10 to 15 per cent of patients presenting with chest trauma require definitive surgical repair [1,17]. Less than 10% of blunt thoracic injuries and 15% of penetrating injuries require surgical intervention [1,2]. However, surgical interventions may be necessary in severe cases [4]. In our cohort, no major thoracic surgical procedures were performed. Most patients admitted to the ICU had blunt trauma and were older, with multiple comorbid conditions. Tube thoracostomy was the main intervention and was used in both the Non-survivor group (25%) and the Survivor group (16.3%).

Treatment with chest tube insertion, pain control, and chest physiotherapy in blunt thoracic trauma yields favorable outcomes in most patients [4]. Optimal treatment with better survival rates can be achieved in specialized centers with multidisciplinary teamwork and thoracic surgery experience [4].

Elderly patients are particularly prone to rib fractures and related complications, with pneumonia rates reaching up to 31% [18]. In cases of three or more rib fractures, the risk of complications such as pulmonary contusion and pneumonia increases [19].

Although rib fractures are significant in thoracic trauma, fractures may also occur in other bone structures in the chest cage. Sternum fractures usually result from blunt trauma. Sternum fractures can cause vascular, pulmonary, and myocardial contusions. Electrocardiography and cardiac enzymes should be evaluated in sternum fractures [19]. In our cohort, thoracic injury patterns were similar between the Survivor group and the Non-survivor group. Mortality appeared to be more closely related to patient age and comorbid conditions than to thoracic injury characteristics alone. Therefore, we believe that the reason why the contribution of thoracic trauma to mortality was not significant in our study is due to the heterogeneity between the groups. We believe that if we had compared similar patient groups without comorbidities, we would have obtained a more meaningful result in this regard.

Contrast-enhanced computed tomography (CT) is the most specific tool in thoracic trauma. It is useful in cases of hemodynamically stable severe trauma and when findings on CXR and/or US are inconclusive. In fact, chest injuries can be detected in 71% of patients with normal chest X-rays, and in 37.5% of these cases, the injuries may require life-saving interventions [3,20]. CT scans are excellent at identifying lung parenchymal damage and provide definitive information about the severity and characteristics of lung injuries. Early CT scans can accurately predict the risk of acute respiratory distress syndrome (ARDS) and indicate that the likelihood of ARDS increases with the volume of the initial lung injury [3]. In our study, chest CT was performed in most patients in both the Survivor group and the Non-survivor group. Acute respiratory distress syndrome was diagnosed more frequently in patients who died. This finding may be related to the extent of initial lung injury detected on CT rather than isolated thoracic injury patterns alone.

The use of validated and appropriate trauma scoring systems is important to ensure timely intervention and improve outcomes. A low GCS score is a strong predictor of mortality in patients with thoracic trauma [19,21]. In our study, low GCS scores were also associated with mortality. We hypothesize that this is because a low GCS score may also be associated with the need for endotracheal intubation and mechanical ventilation.

Trauma scoring systems convert the severity of injury into a number. They enable clinicians to translate different injury severities into a common language [22]. Quantitative characterizations of injuries are essential for the meaningful evaluation of research and patient outcomes, quality improvement, and prevention programs [22,23]. This demonstrates not only the need for such scoring systems, but also their shortcomings in meeting all requirements [22]. Among the three clinical scores used in patients with thoracic trauma (GCS, ISI, and APACHE II), the APACHE II score demonstrated the highest discriminatory performance and was identified as the strongest clinical predictor of mortality. Even after adjusting for important confounding variables like age, gender, serum lactate levels, and the existence of head trauma, our study’s multivariate logistic regression analysis revealed an independent relationship between clinical scores and mortality.

In a study by Beshay et al., the presence of severe lung contusion, high ISS and Abbreviated Injury Scale scores, and advanced age were reported as independent risk factors directly associated with higher mortality rates [4]. In our cohort, the ISS was not associated with mortality. This suggests that anatomical injury alone may not reflect clinical instability in intensive care patients. Therefore, a patient with a low ISS may be in hemorrhagic shock, profound hypotension, or at high risk of death. In our study, the need for vasopressors was found to be significantly different between groups, and we believe this could explain why the ISS score was not significant. Our study’s multivariate analysis results significantly add to discussions in the literature. The APACHE II-based model (Model 3) had the best model fit of the three regression models we developed, with the lowest AIC value (77.61) and the highest explanatory power (Nagelkerke R^2^ (0.548)). This lends credence to the idea that, when it comes to forecasting mortality, scoring systems that concentrate on physiological indicators in thoracic trauma patients under critical care monitoring may be more effective than those that only consider anatomical injuries.

In patients with shock presenting with high lactate levels, fluid resuscitation, vasopressors, or inotropic agents may be used in treatment. Elevated lactate levels may help identify a patient with tissue hypoperfusion that initially masks abnormal vital signs [24]. Elevated lactate levels and lactate clearance rate show a strong correlation with multi-organ dysfunction and survival following traumatic injury [24,25]. These factors also suggest the need for multiorgan dysfunction support systems. In our study, patients who died had higher lactate levels at admission. Hypoperfusion leads to decreased renal perfusion, the development of acute kidney injury (AKI), and the need for dialysis. In our cohort, urea and creatine levels were higher in the Non-survivor group. Secondary to trauma, rhabdomyolysis also triggers the development of AKI. If AKI develops after rhabdomyolysis, mortality can reach 8%. Furthermore, renal ischemia may occur in trauma patients due to concomitant hypovolemia [26]. One option for correcting the acidosis and inflammatory mediators present in AKI is the implementation of CRRT [27]. In our study, laboratory values in the Non-survivor group showed higher lactate, urea, and creatine levels, lower hemoglobin and pH levels, and a more negative BE value. The need for CRRT was also found to be higher in the Non-survivor group.

This study has several limitations. It was conducted at a single center and had a retrospective design. The main limitation of this study is the imbalance between cohort sizes (n = 24 vs. n = 80), which may have limited the statistical power to detect smaller differences between groups and increased the risk of type II error. The number of included patients was relatively limited. In addition, mortality was evaluated as all-cause mortality, and short-term outcomes were not analyzed separately. Patients with major cardiac and vascular injuries were followed in a different ICU and were not included in this study. A further limitation of the study was the inability to calculate the Sequential Organ Failure Assessment score, which is a useful tool for evaluating organ dysfunction, due to a lack of longitudinal data. However, as chronic comorbidities and advanced age were found to be the main causes for mortality in our cohort, APACHE II, which takes these particular factors into account, was seen to be a reliable and suitable substitute for early risk classification. These factors should be considered when interpreting the results.

## 5. Conclusions

Patients with thoracic trauma should be comprehensively assessed as soon as possible and receive urgent intervention. Factors that may shorten the length of ICU stay include infection control, early correction of acidosis, and maintenance of hemodynamic stability, which may reduce mortality. We believe that as the length of stay increases, complications will increase and there will be a need for increased supportive treatments such as mechanical ventilation and CRRT. Concomitant traumatic injuries, comorbidities, high APACHE II scores, and low GCS scores increase mortality. In this cohort, APACHE II was more closely related to overall clinical severity than the other scoring systems. In the APACHE II-based model, multivariate analysis revealed that advanced age (OR: 1.06), increased lactate levels (OR: 1.57), and concomitant head trauma (OR: 9.08) were independent predictors of mortality. Our data indicate that age-related frailty and acute physiological derangement, as best represented by the APACHE II score, are more significant determinants of survival than anatomic injury severity alone. Larger, multicenter prospective studies are needed to further clarify factors affecting outcomes in critically ill patients with thoracic trauma.

## Figures and Tables

**Table 1:** Demographic data, clinical characteristics at admission, trauma and thoracic injury profiles, and laboratory parameters of patients who did not survive (Non-survivors) and those who survived (Survivors).

	Non-Survivors (n = 24, 23.1%)	Survivors (n = 80, 76.9%)	*p*-Value
**Demographic characteristics**			
Age	59.33 ± 22.21	40.50 ± 17.71	<0.001 ^1^
Gender (female/male)	12 (50)/12 (50)	16 (20)/64 (80)	0.008 ^2^
**Clinical findings**			
GCS	6 (3–15)	15 (3–15)	<0.001 ^3^
ISS	27 (22–43)	27 (4–38)	0.075 ^3^
APACHE II	23 (6–76)	12 (0–35)	<0.001 ^3^
Head trauma	20 (83.3)	44 (55)	0.024 ^2^
Skeletal system pathology	8 (33.3)	14 (17.5)	0.167 ^2^
Intubated upon arrival	14 (58.3)	21 (26.2)	0.008 ^2^
TCA	3 (12.5)	0 (0)	0.011 ^4^
Comorbidity	9 (37.5)	8 (10)	0.003 ^4^
Diabetes Mellitus	2 (8.3)	2 (2.5)	0.227 ^4^
Cerebrovascular disease	2 (8.3)	4 (5)	0.620 ^4^
Cardiac disease	3 (12.5)	1 (1.25)	0.038 ^4^
Chronic kidney disease	0 (0)	2 (2.5)	>0.999 ^4^
Hypertension	5 (20.8)	3 (3.75)	0.015 ^4^
Malignancy	2 (8.3)	2 (2.5)	0.227 ^4^
Type of trauma (blunt/penetrating)	24 (100)/0 (0)	78 (97.5)/2 (2.5)	>0.999 ^4^
**Thoracic injuries and related procedures**			
Consultation with a thoracic surgeon	22 (91.7)	61 (76.3)	0.147 ^4^
Chest CT scan	24 (100)	79 (98.8)	>0.999 ^4^
Pneumothorax	8 (33.3)	33 (41.3)	0.647 ^2^
Hemothorax	9 (37.5)	16 (20)	0.137 ^2^
Pneumomediastinum	2 (8.3)	5 (6.3)	0.661 ^4^
Pulmonary contusion	14 (58.3)	50 (62.5)	0.898 ^2^
Scapular fracture	3 (12.5)	10 (12.5)	>0.999 ^4^
Clavicula fracture	4 (16.7)	16 (20)	>0.999 ^4^
Sternal fracture	7 (29.2)	10 (12.5)	0.065 ^4^
Rib fracture	17 (70.8)	48 (60)	0.471 ^2^
Unilateral rib fracture	9 (37.5)	32 (40)	>0.999 ^2^
Bilateral rib fracture	8 (33.3)	16 (20)	0.279 ^2^
Tube thoracostomy	6 (25)	13 (16.3)	0.371 ^4^
Operated on for other injuries	10 (41.7)	33 (41.3)	>0.999 ^2^
**Laboratory Findings**			
Hemoglobin (g/dL)	11.07 ± 1.82	12.78 ± 1.84	<0.001 ^1^
Platelet count (K/uL)	218.88 ± 57.72	221.03 ± 65.55	0.885 ^1^
Urea (mg/dL)	43 [37.25–53]	32 [27–38]	<0.001 ^3^
Creatine (mg/dL)	1.10 [0.97–1.49]	0.90 [0.75–1]	0.002 ^3^
PT (s)	11.5 [10–13.25]	11 [10–12]	0.131 ^3^
aPTT (s)	28 [24.5–30]	26 [23.63–29]	0.225 ^3^
INR	1.20 [1.09–1.29]	1 [1–1.1]	<0.001 ^3^
Calcium (mg/dL)	8 [7.47–8]	8 [8–9]	0.014 ^3^
Ph	7.3 [7.2–7.3]	7.4 [7.3–7.4]	<0.001 ^3^
PaO2	69 [51.25–86.40]	67.5 [52–90]	0.761 ^3^
PCO2	42.36 ± 11.27	39.23 ± 7.76	0.212 ^5^
Lactate	3.10 [2–7.15]	2.15 [1.50–3.92]	0.003 ^3^
Base Deficit	−6.62 ± 5.23	−3.80 ± 3.58	0.003 ^1^

^1^ Student’s *t*-test; ^2^ Chi-square test with Yates continuity correction; ^3^ Mann–Whitney U test; ^4^ Fisher Exact test; ^5^ Welch’s *t*-test. ISS, Injury Severity Score; APACHE II, Acute Physiology and Chronic Health Evaluation II; GCS, Glasgow Coma Scale; TCA, traumatic cardiac arrest; CT, computed tomography; PT, prothrombin time; aPTT, activated partial thromboplastin time; INR, International Normalized Ratio; PaO2, Partial Pressure of Oxygen; PCO2, Partial Pressure of Carbon Dioxide.

**Table 2:** ICU course, outcomes, and complications related to the intensive care process in patients who did not survive (Non-survivors) and those who were discharged from the intensive care unit (Survivors).

	Non-Survivors (n = 24, 23.1%)	Survivors (n = 80, 76.9%)	*p*-Value
MV Requirement	23 (95.8)	24 (30)	<0.001 ^1^
MV Time day	14 (2–242)	11 (2–90)	0.757 ^2^
ICU Length of Stay (Days)	15 (2–242)	7 (2–120)	0.045 ^2^
ASP	1 (4.2)	2 (2.5)	0.549 ^3^
VAP	14 (58.3)	13 (16.3)	<0.001 ^1^
CRRT Requirement	7 (29.2)	2 (2.5)	<0.001 ^3^
VP Requirement	18 (75)	23 (28.8)	<0.001 ^1^
ARDS	7 (29.2)	2 (2.5)	<0.001 ^3^

^1^ Chi-square test with Yates continuity correction; ^2^ Mann–Whitney U test; ^3^ Fisher Exact test. Data were presented as mean ± standard deviation, median (ranges: min–max) or median with quartiles [Q1–Q3] for numerical variables, as appropriate. Categorical variables were also described as count (n) and percentage (%). MV, mechanical ventilation; ICU, intensive care unit; ASP, aspiration pneumonia; VAP, ventilator-associated pneumonia; CRRT, continuous renal replacement therapy requirement; VP, vasopressor; ARDS, acute respiratory distress syndrome.

**Table 3:** ROC analysis results regarding the predictive performance of the Glasgow Coma Scale, ISS, and APACHE II scores for mortality.

	Glasgow Coma Scale	Injury Severity Score	APACHE II Score
ROC curve statistics			
AUC (95% CI)	0.706 (0.592–0.820)	0.612 (0.512–0.712)	0.751 (0.630–0.872)
*p*-value	<0.001	0.028	<0.001
Cut-off point	12.5	23.5	14.5
Diagnostic measures			
Sensitivity (95% CI)	70.83 (48.91–87.38)	91.67 (73–98.97)	79.17 (57.85–92.87)
Specificity (95% CI)	68.75 (57.41–78.65)	36.25 (25.79–47.76)	61.25 (49.70–71.94)
PPV (95% CI)	40.48 (31.01–50.71)	30.14 (26.01–34.61)	38 (30.30–46.36)
NPV (95% CI)	88.71 (80.54–93.72)	93.55 (78.85–98.26)	90.74 (81.51–95.61)

APACHE II, Acute Physiology and Chronic Health Evaluation II; ROC, Receiver Operating Characteristic; AUC, area under the curve; PPV, positive predictive value; NPV, negative predictive value.

**Table 4:** Multiple analysis.

	Multiple Logistic Reg. Analysis		Model Diagnostic Performance					
	OR [95% CI]	*p*-Value	Acc.	Spec.	Sens.	AUC	Nagelkerke *R*^2^	AIC
**Model–1**			90.4%	96.3%	70.8%	0.887	0.544	78.06
Age (years)	1.07 [1.03–1.11]	<0.001						
Sex (ref: male)	2.99 [0.81–11.11]	0.101						
Lactate	1.64 [1.12–2.39]	0.011						
Head trauma	5.45 [1.14–26.06]	0.034						
Glasgow Coma Score	0.89 [0.79–1.00]	0.056						
**Model–2**			85.6%	93.8%	58.3%	0.898	0.538	78.73
Age (years)	1.07 [1.03–1.12]	<0.001						
Sex (ref: male)	2.26 [0.65–7.91]	0.200						
Lactate	1.89 [1.26–2.83]	0.002						
Head trauma	3.81 [0.73–19.93]	0.113						
Injury Severity Score	1.14 [0.98–1.34]	0.095						
**Model–3**			88.5%	96.3%	62.5%	0.894	0.548	77.61
Age (years)	1.06 [1.02–1.10]	0.002						
Sex (ref: male)	2.58 [0.71–9.34]	0.148						
Lactate	1.57 [1.05–2.34]	0.027						
Head trauma	9.08 [1.75–47.16]	0.009						
APACHE II Score	1.07 [1.00–1.15]	0.045						

Reg, regression; Ref, reference; Acc, accuracy; Spec, specificity; Sens. sensitivity; AUC, area under the curve; APACHE II, Acute Physiology and Chronic Health Evaluation II.

## Data Availability

The original contributions presented in the study are included in the article; further inquiries can be directed to the corresponding author.
